# *N*-(9,9-Dipropyl-9*H*-fluoren-2-yl)-7-(piperidin-1-yl)-2,1,3-benzothia­diazol-4-amine

**DOI:** 10.1107/S1600536812008239

**Published:** 2012-02-29

**Authors:** M. N. K. Prasad Bolisetty, K. R. Justin Thomas, Seik Weng Ng, Edward R. T. Tiekink

**Affiliations:** a Organic Materials Laboratory, Department of Chemistry, Indian Institute of Technology Roorkee, Roorkee 247 667, India; b Department of Chemistry, University of Malaya, 50603 Kuala Lumpur, Malaysia; c Chemistry Department, Faculty of, Science, King Abdulaziz University, PO Box 80203 Jeddah, Saudi Arabia

## Abstract

In the title compound, C_30_H_34_N_4_S, each of the benzothia­diazole and fluorene fused ring systems is almost planar (r.m.s. deviations = 0.010 and 0.013 Å, respectively) and they are inclined to each other with a dihedral angle of 61.69 (3)°; the S atom is directed away from the rest of the mol­ecule. Each of the benzothiadiazole ring N atoms forms a significant intra­molecular contact, *i.e*. N—H⋯N or C—H⋯N. In the crystal, linear supra­molecular chains along the *c* axis are generated by C—H⋯N inter­actions involving the tertiary amine N atom.

## Related literature

For the application of benzo[*c*][1,2,5]thia­diazole-based polymers and small mol­ecules in organic light-emitting diodes and bulk heterojunction solar cells, see: Beaujuge *et al.* (2012 ▶); Horie *et al.* (2012 ▶); Thomas *et al.* (2004 ▶, 2008 ▶). For related structures, see: Sakurai *et al.* (2005 ▶); Chen *et al.* (2010 ▶); Tao *et al.* (2011 ▶).
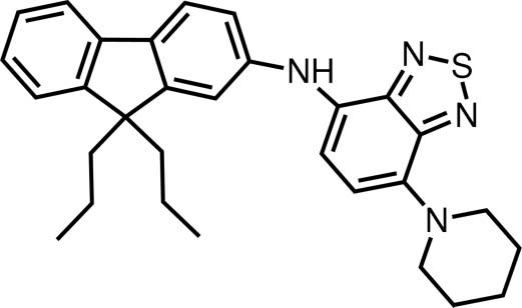


## Experimental

### 

#### Crystal data

C_30_H_34_N_4_S
*M* *_r_* = 482.67
Monoclinic, 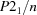

*a* = 9.6111 (1) Å
*b* = 21.9632 (2) Å
*c* = 12.6954 (1) Å
β = 103.936 (1)°
*V* = 2601.00 (4) Å^3^
*Z* = 4
Cu *K*α radiation
μ = 1.29 mm^−1^
*T* = 100 K
0.35 × 0.20 × 0.05 mm

#### Data collection

Agilent SuperNova Dual diffractometer with an Atlas detector
Absorption correction: multi-scan (*CrysAlis PRO*; Agilent, 2010 ▶) *T*_min_ = 0.302, *T*_max_ = 1.000
14383 measured reflections
5430 independent reflections
4961 reflections with *I* > 2σ(*I*)
*R*_int_ = 0.023

#### Refinement

*R*[*F* ^2^ > 2σ(*F* ^2^)] = 0.038
*wR*(*F* ^2^) = 0.106
*S* = 1.03
5430 reflections
317 parameters
H-atom parameters constrained
Δρ_max_ = 0.38 e Å^−3^
Δρ_min_ = −0.37 e Å^−3^

### 

Data collection: *CrysAlis PRO* (Agilent, 2010 ▶); cell refinement: *CrysAlis PRO*; data reduction: *CrysAlis PRO*; program(s) used to solve structure: *SHELXS97* (Sheldrick, 2008 ▶); program(s) used to refine structure: *SHELXL97* (Sheldrick, 2008 ▶); molecular graphics: *ORTEP-3* (Farrugia, 1997 ▶) and *DIAMOND* (Brandenburg, 2006 ▶); software used to prepare material for publication: *publCIF* (Westrip, 2010 ▶).

## Supplementary Material

Crystal structure: contains datablock(s) global, I. DOI: 10.1107/S1600536812008239/hg5183sup1.cif

Structure factors: contains datablock(s) I. DOI: 10.1107/S1600536812008239/hg5183Isup2.hkl

Supplementary material file. DOI: 10.1107/S1600536812008239/hg5183Isup3.cml

Additional supplementary materials:  crystallographic information; 3D view; checkCIF report

## Figures and Tables

**Table 1:** Hydrogen-bond geometry (Å, °)

*D*—H⋯*A*	*D*—H	H⋯*A*	*D*⋯*A*	*D*—H⋯*A*
N4—H4⋯N2	0.88	2.55	2.8567 (17)	101
C5—H5*B*⋯N1	0.99	2.60	3.212 (2)	120
C20—H20⋯N3^i^	0.95	2.55	3.4577 (16)	161

Symmetry code: (i) 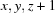.

**Fig. 1.**
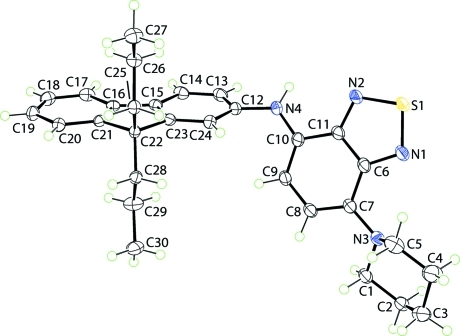
The molecular structure of (I) showing the atom-labelling scheme and displacement ellipsoids at the 50% probability level.

**Fig. 2.**
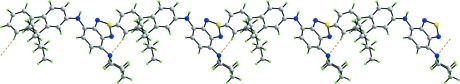
A view of the linear supramolecular chain along the c axis in (I). The C—H···N interactions are shown as orange dashed lines.

## supplementary crystallographic information

### Comment

Benzo[*c*][1,2,5]thiadiazole-based polymers (Beaujuge *et al.*, 2012; Horie *et al.*, 2012) and small molecules (Thomas *et al.*, 2004; Thomas *et al.*, 2008) have been explored as functional materials for applications in organic light-emitting diodes and bulk heterojunction solar cells. A new diarylamine, *N*-(9,9-dipropyl-9*H*-fluoren-2-yl)-7-(piperidin-1-yl)benzo[*c*][1,2,5]thiadiazol-4-amine (I), featuring benzo[*c*][1,2,5]thiadiazole and fluorene units has been synthesized as a building block for the development of organic dyes suitable for use as sensitizers in dye-sensitized solar cells. Herein, the crystal and molecular structure of (I) is described. Related structures are known (Sakurai *et al.*, 2005; Chen *et al.*, 2010; Tao *et al.*, 2011).

In (I), Fig. 1, each of the benzothiadiazole (r.m.s. deviation = 0.010 Å) and fluorene (0.013 Å) fused ring systems are planar and these are inclined at 61.69 (3)° so that the S atom is directed away from the rest of the molecule. Each of the *n*-propyl groups connected at the C22 atom adopt open conformations with the C22—C25—C26—C27 and C22–C28—C29—C30 torsion angles being -179.98 (10) and 179.50 (11)°, respectively. Each of the ring N atoms forms a significant intramolecular contact, N1 with C5—*H* and N2 with N4—*H*, Table 1. The most notable intermolecular contact is of the type C—H···N involving the tertiary amine-N atom, Table 1. These lead to the formation of linear supramolecular chains along the *c* axis.

### Experimental

A mixture of 9,9-dipropyl-9*H*-fluoren-2-amine (0.66 g, 2.5 mmol), 4-bromo-7-(piperidin-1-yl)benzo[*c*][1,2,5]thiadiazole (0.75 g, 2.5 mmol), Pd(dba)_2_ (dba = (1*E*,4*E*)-1,5-diphenylpenta-1,4-dien-3-one; 0.03 mmol), dppf (1,1'-bis(diphenylphosphino)ferrocene; 0.03 mmol), *t*-BuONa (0.38 g, 3 mmol) and toluene (15 ml) was taken in a Schlenk tube and heated at 353 K with stirring for 48 h. After completion of the reaction, the volatiles were evaporated to leave a pink residue. The residue was purified by column chromatography on silica gel by using a dichloromethane/hexanes mixture (1:2) as eluent. Yield 1.1 g (91%). *M*.pt. 393–395 K. Crystals were grown from its solution in a dichloromethane/hexanes mixture.

^1^H NMR (CDCl_3_, 500.13 MHz) δ p.p.m.: 0.65–0.70 (m, 10 H), 1.65–1.70 (m, 2 H), 1.86–1.95 (m, 8 H), 3.3 (t, J = 5.5 Hz, 4 H), 6.78 (d, J = 8.5 Hz, 1 H), 6.92 (s, 1 H), 7.17 (d, J = 8 Hz, 1 H), 7.23–7.25 (m, 3 H), 7.30–7.33 (m, 2 H), 7.63 (t, J = 8.5 Hz, 2 H). ^13^C NMR (CDCl_3_, 125.77 MHz) δ p.p.m.: 14.6, 17.3, 24.6, 26.2, 43.0, 52.5, 55.3, 107.4, 113.5, 114.6, 117.5, 119.0, 120.5, 122.8, 126.2, 126.8, 130.0, 135.3, 138.0, 140.9, 141.1,150.0, 150.2, 150.8, 152.4.

### Refinement

Carbon-bound H-atoms were placed in calculated positions [N—H = 0.88 Å; C—H 0.95 to 0.99 Å, *U*_iso_(H) 1.2 to 1.5*U*_eq_(N,*C*)] and were included in the refinement in the riding model approximation.

### Crystal data

**Table:**

C_30_H_34_N_4_S	*F*(000) = 1032
*M**_r_* = 482.67	*D*_x_ = 1.233 Mg m^−^^3^
Monoclinic, *P*2_1_/*n*	Cu *K*α radiation, λ = 1.54184 Å
Hall symbol: -P 2yn	Cell parameters from 8442 reflections
*a* = 9.6111 (1) Å	θ = 3.6–76.4°
*b* = 21.9632 (2) Å	µ = 1.29 mm^−^^1^
*c* = 12.6954 (1) Å	*T* = 100 K
β = 103.936 (1)°	Block, brown
*V* = 2601.00 (4) Å^3^	0.35 × 0.20 × 0.05 mm
*Z* = 4	

### Data collection

**Table:**

Agilent SuperNova Dual diffractometer with an Atlas detector	5430 independent reflections
Radiation source: SuperNova (Cu) X-ray Source	4961 reflections with *I* > 2σ(*I*)
Mirror monochromator	*R*_int_ = 0.023
Detector resolution: 10.4041 pixels mm^-1^	θ_max_ = 76.6°, θ_min_ = 4.0°
ω scan	*h* = −12→12
Absorption correction: multi-scan (*CrysAlis PRO*; Agilent, 2010)	*k* = −27→18
*T*_min_ = 0.302, *T*_max_ = 1.000	*l* = −14→15
14383 measured reflections	

### Refinement

**Table:**

Refinement on *F*^2^	Secondary atom site location: difference Fourier map
Least-squares matrix: full	Hydrogen site location: inferred from neighbouring sites
*R*[*F*^2^ > 2σ(*F*^2^)] = 0.038	H-atom parameters constrained
*wR*(*F*^2^) = 0.106	*w* = 1/[σ^2^(*F*_o_^2^) + (0.0594*P*)^2^ + 0.8093*P*] where *P* = (*F*_o_^2^ + 2*F*_c_^2^)/3
*S* = 1.03	(Δ/σ)_max_ < 0.001
5430 reflections	Δρ_max_ = 0.38 e Å^−^^3^
317 parameters	Δρ_min_ = −0.37 e Å^−^^3^
0 restraints	Extinction correction: *SHELXL97* (Sheldrick, 2008), Fc^*^=kFc[1+0.001xFc^2^λ^3^/sin(2θ)]^-1/4^
Primary atom site location: structure-invariant direct methods	Extinction coefficient: 0.00114 (17)

### Special details

**Table:**

Geometry. All e.s.d.'s (except the e.s.d. in the dihedral angle between two l.s. planes) are estimated using the full covariance matrix. The cell e.s.d.'s are taken into account individually in the estimation of e.s.d.'s in distances, angles and torsion angles; correlations between e.s.d.'s in cell parameters are only used when they are defined by crystal symmetry. An approximate (isotropic) treatment of cell e.s.d.'s is used for estimating e.s.d.'s involving l.s. planes.
Refinement. Refinement of *F*^2^ against ALL reflections. The weighted *R*-factor *wR* and goodness of fit *S* are based on *F*^2^, conventional *R*-factors *R* are based on *F*, with *F* set to zero for negative *F*^2^. The threshold expression of *F*^2^ > σ(*F*^2^) is used only for calculating *R*-factors(gt) *etc*. and is not relevant to the choice of reflections for refinement. *R*-factors based on *F*^2^ are statistically about twice as large as those based on *F*, and *R*- factors based on ALL data will be even larger.

### Fractional atomic coordinates and isotropic or equivalent isotropic displacement parameters (Å^2^)

**Table:**

	*x*	*y*	*z*	*U*_iso_*/*U*_eq_	
S1	0.17524 (4)	0.516536 (18)	0.29347 (3)	0.03740 (12)	
N1	0.32500 (14)	0.48122 (5)	0.30043 (9)	0.0300 (3)	
N2	0.12335 (14)	0.48521 (6)	0.39313 (9)	0.0337 (3)	
N3	0.56133 (11)	0.39374 (5)	0.35759 (8)	0.0218 (2)	
N4	0.10144 (13)	0.40732 (6)	0.56862 (9)	0.0314 (3)	
H4	0.0231	0.4244	0.5295	0.038*	
C1	0.63792 (15)	0.33572 (6)	0.36929 (10)	0.0259 (3)	
H1A	0.5693	0.3018	0.3666	0.031*	
H1B	0.7085	0.3343	0.4403	0.031*	
C2	0.71468 (16)	0.32872 (7)	0.27814 (11)	0.0337 (3)	
H2A	0.7681	0.2898	0.2871	0.040*	
H2B	0.6431	0.3276	0.2075	0.040*	
C3	0.81786 (18)	0.38097 (8)	0.27843 (16)	0.0464 (4)	
H3A	0.8604	0.3774	0.2150	0.056*	
H3B	0.8965	0.3792	0.3450	0.056*	
C4	0.73966 (18)	0.44147 (8)	0.27394 (16)	0.0452 (4)	
H4A	0.6711	0.4458	0.2024	0.054*	
H4B	0.8098	0.4752	0.2821	0.054*	
C5	0.65895 (15)	0.44563 (6)	0.36334 (13)	0.0329 (3)	
H5A	0.7283	0.4461	0.4350	0.039*	
H5B	0.6035	0.4840	0.3555	0.039*	
C6	0.34160 (15)	0.44213 (6)	0.38424 (10)	0.0240 (3)	
C7	0.45773 (14)	0.39993 (5)	0.41925 (9)	0.0216 (2)	
C8	0.45092 (14)	0.36470 (6)	0.50703 (10)	0.0239 (3)	
H8	0.5275	0.3373	0.5344	0.029*	
C9	0.33591 (15)	0.36683 (6)	0.55952 (10)	0.0254 (3)	
H9	0.3385	0.3406	0.6195	0.031*	
C10	0.22113 (14)	0.40543 (6)	0.52650 (10)	0.0256 (3)	
C11	0.22551 (15)	0.44449 (6)	0.43700 (10)	0.0259 (3)	
C12	0.09800 (14)	0.38303 (6)	0.67210 (10)	0.0249 (3)	
C13	−0.01831 (14)	0.34774 (6)	0.68202 (10)	0.0266 (3)	
H13	−0.0910	0.3381	0.6191	0.032*	
C14	−0.02985 (13)	0.32626 (6)	0.78291 (10)	0.0237 (3)	
H14	−0.1099	0.3024	0.7892	0.028*	
C15	0.07814 (13)	0.34037 (5)	0.87438 (10)	0.0193 (2)	
C16	0.09581 (12)	0.32574 (5)	0.98970 (10)	0.0184 (2)	
C17	0.00960 (13)	0.29269 (5)	1.04300 (11)	0.0233 (3)	
H17	−0.0776	0.2749	1.0036	0.028*	
C18	0.05390 (14)	0.28621 (6)	1.15523 (11)	0.0263 (3)	
H18	−0.0035	0.2636	1.1926	0.032*	
C19	0.18115 (14)	0.31241 (6)	1.21333 (10)	0.0246 (3)	
H19	0.2095	0.3076	1.2899	0.030*	
C20	0.26771 (13)	0.34567 (5)	1.16012 (10)	0.0209 (2)	
H20	0.3546	0.3636	1.1997	0.025*	
C21	0.22408 (12)	0.35202 (5)	1.04844 (9)	0.0173 (2)	
C22	0.29987 (12)	0.38581 (5)	0.97382 (9)	0.0169 (2)	
C23	0.19643 (12)	0.37525 (5)	0.86392 (9)	0.0185 (2)	
C24	0.20788 (13)	0.39649 (6)	0.76383 (10)	0.0225 (2)	
H24	0.2887	0.4198	0.7573	0.027*	
C25	0.31877 (12)	0.45412 (5)	1.00244 (9)	0.0190 (2)	
H25A	0.3827	0.4579	1.0760	0.023*	
H25B	0.3676	0.4737	0.9510	0.023*	
C26	0.18116 (13)	0.48892 (5)	0.99994 (10)	0.0221 (2)	
H26A	0.1318	0.4701	1.0518	0.026*	
H26B	0.1167	0.4859	0.9264	0.026*	
C27	0.20981 (15)	0.55594 (6)	1.02916 (12)	0.0281 (3)	
H27A	0.1187	0.5768	1.0257	0.042*	
H27B	0.2581	0.5748	0.9777	0.042*	
H27C	0.2710	0.5592	1.1028	0.042*	
C28	0.44887 (12)	0.35842 (5)	0.97719 (9)	0.0200 (2)	
H28A	0.4883	0.3788	0.9212	0.024*	
H28B	0.5134	0.3678	1.0487	0.024*	
C29	0.45097 (15)	0.29009 (6)	0.95892 (13)	0.0325 (3)	
H29A	0.4132	0.2692	1.0154	0.039*	
H29B	0.3866	0.2803	0.8875	0.039*	
C30	0.59965 (16)	0.26589 (7)	0.96215 (12)	0.0354 (3)	
H30A	0.5946	0.2219	0.9492	0.053*	
H30B	0.6632	0.2742	1.0335	0.053*	
H30C	0.6372	0.2859	0.9058	0.053*	

### Atomic displacement parameters (Å^2^)

**Table:**

	*U*^11^	*U*^22^	*U*^33^	*U*^12^	*U*^13^	*U*^23^
S1	0.0515 (2)	0.0408 (2)	0.02084 (18)	0.02678 (16)	0.01052 (15)	0.01025 (13)
N1	0.0437 (7)	0.0260 (6)	0.0198 (5)	0.0138 (5)	0.0064 (5)	0.0039 (4)
N2	0.0431 (7)	0.0410 (7)	0.0160 (5)	0.0213 (6)	0.0053 (5)	0.0044 (5)
N3	0.0262 (5)	0.0178 (5)	0.0204 (5)	0.0018 (4)	0.0034 (4)	0.0002 (4)
N4	0.0323 (6)	0.0459 (7)	0.0150 (5)	0.0160 (5)	0.0039 (4)	0.0050 (5)
C1	0.0323 (7)	0.0230 (6)	0.0215 (6)	0.0071 (5)	0.0045 (5)	0.0025 (5)
C2	0.0393 (8)	0.0370 (8)	0.0259 (6)	0.0193 (6)	0.0101 (6)	0.0082 (6)
C3	0.0343 (8)	0.0523 (10)	0.0577 (10)	0.0167 (7)	0.0210 (7)	0.0232 (8)
C4	0.0334 (8)	0.0388 (8)	0.0674 (11)	0.0045 (6)	0.0203 (8)	0.0206 (8)
C5	0.0298 (7)	0.0234 (6)	0.0419 (8)	−0.0027 (5)	0.0018 (6)	0.0014 (6)
C6	0.0351 (7)	0.0203 (6)	0.0152 (5)	0.0060 (5)	0.0030 (5)	−0.0016 (4)
C7	0.0287 (6)	0.0177 (5)	0.0164 (5)	0.0031 (5)	0.0017 (5)	−0.0027 (4)
C8	0.0295 (6)	0.0209 (6)	0.0191 (6)	0.0064 (5)	0.0020 (5)	0.0006 (4)
C9	0.0338 (7)	0.0249 (6)	0.0169 (5)	0.0068 (5)	0.0048 (5)	0.0025 (5)
C10	0.0319 (7)	0.0295 (6)	0.0140 (5)	0.0076 (5)	0.0030 (5)	−0.0022 (5)
C11	0.0336 (7)	0.0276 (6)	0.0142 (5)	0.0103 (5)	0.0012 (5)	−0.0019 (5)
C12	0.0279 (6)	0.0302 (6)	0.0159 (6)	0.0100 (5)	0.0036 (5)	0.0000 (5)
C13	0.0251 (6)	0.0282 (6)	0.0213 (6)	0.0058 (5)	−0.0043 (5)	−0.0059 (5)
C14	0.0205 (6)	0.0211 (6)	0.0266 (6)	0.0010 (5)	−0.0002 (5)	−0.0039 (5)
C15	0.0191 (5)	0.0165 (5)	0.0218 (6)	0.0023 (4)	0.0038 (4)	−0.0016 (4)
C16	0.0179 (5)	0.0146 (5)	0.0229 (6)	0.0015 (4)	0.0052 (4)	−0.0008 (4)
C17	0.0198 (6)	0.0177 (5)	0.0331 (7)	−0.0016 (4)	0.0079 (5)	0.0010 (5)
C18	0.0280 (6)	0.0217 (6)	0.0336 (7)	−0.0005 (5)	0.0161 (5)	0.0058 (5)
C19	0.0297 (6)	0.0248 (6)	0.0216 (6)	0.0018 (5)	0.0105 (5)	0.0047 (5)
C20	0.0223 (6)	0.0216 (6)	0.0192 (6)	−0.0003 (4)	0.0058 (4)	0.0005 (4)
C21	0.0183 (5)	0.0156 (5)	0.0192 (5)	0.0008 (4)	0.0068 (4)	0.0001 (4)
C22	0.0170 (5)	0.0195 (5)	0.0143 (5)	−0.0011 (4)	0.0043 (4)	0.0006 (4)
C23	0.0178 (5)	0.0195 (5)	0.0173 (5)	0.0022 (4)	0.0028 (4)	−0.0015 (4)
C24	0.0229 (6)	0.0267 (6)	0.0183 (6)	0.0031 (5)	0.0054 (5)	0.0003 (5)
C25	0.0185 (5)	0.0198 (5)	0.0189 (5)	−0.0026 (4)	0.0048 (4)	0.0004 (4)
C26	0.0208 (6)	0.0191 (6)	0.0266 (6)	−0.0013 (4)	0.0064 (5)	0.0003 (4)
C27	0.0285 (7)	0.0197 (6)	0.0383 (7)	−0.0012 (5)	0.0124 (5)	0.0001 (5)
C28	0.0168 (5)	0.0254 (6)	0.0177 (5)	−0.0007 (4)	0.0041 (4)	−0.0008 (4)
C29	0.0248 (7)	0.0278 (7)	0.0446 (8)	0.0025 (5)	0.0079 (6)	−0.0072 (6)
C30	0.0330 (7)	0.0407 (8)	0.0333 (7)	0.0122 (6)	0.0097 (6)	−0.0062 (6)

### Geometric parameters (Å, º)

**Table:**

S1—N1	1.6186 (12)	C14—H14	0.9500
S1—N2	1.6208 (13)	C15—C23	1.4035 (17)
N1—C6	1.3464 (16)	C15—C16	1.4683 (16)
N2—C11	1.3455 (16)	C16—C17	1.3934 (17)
N3—C7	1.4136 (16)	C16—C21	1.4023 (16)
N3—C1	1.4611 (15)	C17—C18	1.3926 (19)
N3—C5	1.4668 (17)	C17—H17	0.9500
N4—C10	1.3811 (18)	C18—C19	1.3916 (19)
N4—C12	1.4258 (16)	C18—H18	0.9500
N4—H4	0.8800	C19—C20	1.3974 (17)
C1—C2	1.5225 (18)	C19—H19	0.9500
C1—H1A	0.9900	C20—C21	1.3854 (16)
C1—H1B	0.9900	C20—H20	0.9500
C2—C3	1.516 (2)	C21—C22	1.5202 (15)
C2—H2A	0.9900	C22—C23	1.5235 (15)
C2—H2B	0.9900	C22—C28	1.5443 (16)
C3—C4	1.521 (2)	C22—C25	1.5441 (15)
C3—H3A	0.9900	C23—C24	1.3826 (17)
C3—H3B	0.9900	C24—H24	0.9500
C4—C5	1.524 (2)	C25—C26	1.5212 (16)
C4—H4A	0.9900	C25—H25A	0.9900
C4—H4B	0.9900	C25—H25B	0.9900
C5—H5A	0.9900	C26—C27	1.5269 (17)
C5—H5B	0.9900	C26—H26A	0.9900
C6—C11	1.4339 (19)	C26—H26B	0.9900
C6—C7	1.4365 (17)	C27—H27A	0.9800
C7—C8	1.3710 (17)	C27—H27B	0.9800
C8—C9	1.4223 (19)	C27—H27C	0.9800
C8—H8	0.9500	C28—C29	1.5195 (18)
C9—C10	1.3742 (18)	C28—H28A	0.9900
C9—H9	0.9500	C28—H28B	0.9900
C10—C11	1.4326 (18)	C29—C30	1.5159 (18)
C12—C13	1.391 (2)	C29—H29A	0.9900
C12—C24	1.4023 (17)	C29—H29B	0.9900
C13—C14	1.3943 (19)	C30—H30A	0.9800
C13—H13	0.9500	C30—H30B	0.9800
C14—C15	1.3933 (16)	C30—H30C	0.9800
			
N1—S1—N2	101.00 (6)	C23—C15—C16	108.18 (10)
C6—N1—S1	106.45 (10)	C17—C16—C21	120.38 (11)
C11—N2—S1	105.91 (10)	C17—C16—C15	131.21 (11)
C7—N3—C1	115.62 (10)	C21—C16—C15	108.41 (10)
C7—N3—C5	115.16 (10)	C18—C17—C16	118.62 (11)
C1—N3—C5	111.84 (11)	C18—C17—H17	120.7
C10—N4—C12	123.49 (11)	C16—C17—H17	120.7
C10—N4—H4	118.3	C19—C18—C17	120.88 (11)
C12—N4—H4	118.3	C19—C18—H18	119.6
N3—C1—C2	109.59 (10)	C17—C18—H18	119.6
N3—C1—H1A	109.8	C18—C19—C20	120.63 (12)
C2—C1—H1A	109.8	C18—C19—H19	119.7
N3—C1—H1B	109.8	C20—C19—H19	119.7
C2—C1—H1B	109.8	C21—C20—C19	118.60 (11)
H1A—C1—H1B	108.2	C21—C20—H20	120.7
C3—C2—C1	110.98 (13)	C19—C20—H20	120.7
C3—C2—H2A	109.4	C20—C21—C16	120.89 (11)
C1—C2—H2A	109.4	C20—C21—C22	127.92 (11)
C3—C2—H2B	109.4	C16—C21—C22	111.19 (10)
C1—C2—H2B	109.4	C21—C22—C23	101.04 (9)
H2A—C2—H2B	108.0	C21—C22—C28	111.89 (9)
C2—C3—C4	110.13 (13)	C23—C22—C28	111.24 (9)
C2—C3—H3A	109.6	C21—C22—C25	112.00 (9)
C4—C3—H3A	109.6	C23—C22—C25	112.17 (9)
C2—C3—H3B	109.6	C28—C22—C25	108.43 (9)
C4—C3—H3B	109.6	C24—C23—C15	121.11 (11)
H3A—C3—H3B	108.1	C24—C23—C22	127.70 (11)
C3—C4—C5	111.10 (13)	C15—C23—C22	111.17 (10)
C3—C4—H4A	109.4	C23—C24—C12	118.77 (12)
C5—C4—H4A	109.4	C23—C24—H24	120.6
C3—C4—H4B	109.4	C12—C24—H24	120.6
C5—C4—H4B	109.4	C26—C25—C22	115.52 (9)
H4A—C4—H4B	108.0	C26—C25—H25A	108.4
N3—C5—C4	110.52 (12)	C22—C25—H25A	108.4
N3—C5—H5A	109.5	C26—C25—H25B	108.4
C4—C5—H5A	109.5	C22—C25—H25B	108.4
N3—C5—H5B	109.5	H25A—C25—H25B	107.5
C4—C5—H5B	109.5	C25—C26—C27	111.98 (10)
H5A—C5—H5B	108.1	C25—C26—H26A	109.2
N1—C6—C11	112.87 (11)	C27—C26—H26A	109.2
N1—C6—C7	126.25 (12)	C25—C26—H26B	109.2
C11—C6—C7	120.85 (11)	C27—C26—H26B	109.2
C8—C7—N3	125.01 (11)	H26A—C26—H26B	107.9
C8—C7—C6	115.47 (12)	C26—C27—H27A	109.5
N3—C7—C6	119.28 (11)	C26—C27—H27B	109.5
C7—C8—C9	123.84 (12)	H27A—C27—H27B	109.5
C7—C8—H8	118.1	C26—C27—H27C	109.5
C9—C8—H8	118.1	H27A—C27—H27C	109.5
C10—C9—C8	122.32 (12)	H27B—C27—H27C	109.5
C10—C9—H9	118.8	C29—C28—C22	115.26 (10)
C8—C9—H9	118.8	C29—C28—H28A	108.5
C9—C10—N4	125.65 (12)	C22—C28—H28A	108.5
C9—C10—C11	115.89 (12)	C29—C28—H28B	108.5
N4—C10—C11	118.38 (12)	C22—C28—H28B	108.5
N2—C11—C10	124.64 (13)	H28A—C28—H28B	107.5
N2—C11—C6	113.77 (12)	C30—C29—C28	112.90 (12)
C10—C11—C6	121.58 (11)	C30—C29—H29A	109.0
C13—C12—C24	120.22 (12)	C28—C29—H29A	109.0
C13—C12—N4	119.31 (12)	C30—C29—H29B	109.0
C24—C12—N4	120.42 (13)	C28—C29—H29B	109.0
C12—C13—C14	121.01 (11)	H29A—C29—H29B	107.8
C12—C13—H13	119.5	C29—C30—H30A	109.5
C14—C13—H13	119.5	C29—C30—H30B	109.5
C15—C14—C13	118.84 (12)	H30A—C30—H30B	109.5
C15—C14—H14	120.6	C29—C30—H30C	109.5
C13—C14—H14	120.6	H30A—C30—H30C	109.5
C14—C15—C23	120.02 (11)	H30B—C30—H30C	109.5
C14—C15—C16	131.79 (11)		
			
N2—S1—N1—C6	−0.18 (11)	C14—C15—C16—C17	0.5 (2)
N1—S1—N2—C11	0.05 (11)	C23—C15—C16—C17	179.88 (12)
C7—N3—C1—C2	164.99 (11)	C14—C15—C16—C21	−179.12 (12)
C5—N3—C1—C2	−60.58 (14)	C23—C15—C16—C21	0.29 (13)
N3—C1—C2—C3	57.96 (15)	C21—C16—C17—C18	−0.27 (17)
C1—C2—C3—C4	−54.55 (18)	C15—C16—C17—C18	−179.82 (12)
C2—C3—C4—C5	53.0 (2)	C16—C17—C18—C19	0.35 (18)
C7—N3—C5—C4	−165.85 (11)	C17—C18—C19—C20	−0.24 (19)
C1—N3—C5—C4	59.49 (14)	C18—C19—C20—C21	0.04 (18)
C3—C4—C5—N3	−55.19 (18)	C19—C20—C21—C16	0.05 (17)
S1—N1—C6—C11	0.25 (14)	C19—C20—C21—C22	−179.99 (11)
S1—N1—C6—C7	178.26 (10)	C17—C16—C21—C20	0.07 (17)
C1—N3—C7—C8	18.27 (17)	C15—C16—C21—C20	179.71 (10)
C5—N3—C7—C8	−114.65 (14)	C17—C16—C21—C22	−179.90 (10)
C1—N3—C7—C6	−155.84 (11)	C15—C16—C21—C22	−0.26 (13)
C5—N3—C7—C6	71.24 (14)	C20—C21—C22—C23	−179.84 (11)
N1—C6—C7—C8	−179.99 (12)	C16—C21—C22—C23	0.13 (12)
C11—C6—C7—C8	−2.13 (17)	C20—C21—C22—C28	61.72 (15)
N1—C6—C7—N3	−5.34 (19)	C16—C21—C22—C28	−118.31 (11)
C11—C6—C7—N3	172.53 (11)	C20—C21—C22—C25	−60.27 (15)
N3—C7—C8—C9	−172.02 (12)	C16—C21—C22—C25	119.70 (10)
C6—C7—C8—C9	2.29 (18)	C14—C15—C23—C24	0.43 (17)
C7—C8—C9—C10	−0.6 (2)	C16—C15—C23—C24	−179.06 (10)
C8—C9—C10—N4	175.53 (13)	C14—C15—C23—C22	179.29 (10)
C8—C9—C10—C11	−1.22 (19)	C16—C15—C23—C22	−0.20 (13)
C12—N4—C10—C9	19.6 (2)	C21—C22—C23—C24	178.82 (11)
C12—N4—C10—C11	−163.75 (13)	C28—C22—C23—C24	−62.27 (15)
S1—N2—C11—C10	−178.62 (11)	C25—C22—C23—C24	59.37 (15)
S1—N2—C11—C6	0.09 (15)	C21—C22—C23—C15	0.05 (12)
C9—C10—C11—N2	179.92 (13)	C28—C22—C23—C15	118.96 (11)
N4—C10—C11—N2	2.9 (2)	C25—C22—C23—C15	−119.39 (11)
C9—C10—C11—C6	1.30 (19)	C15—C23—C24—C12	0.54 (18)
N4—C10—C11—C6	−175.70 (12)	C22—C23—C24—C12	−178.11 (11)
N1—C6—C11—N2	−0.24 (17)	C13—C12—C24—C23	−1.47 (18)
C7—C6—C11—N2	−178.37 (11)	N4—C12—C24—C23	176.02 (11)
N1—C6—C11—C10	178.52 (12)	C21—C22—C25—C26	−57.65 (13)
C7—C6—C11—C10	0.39 (19)	C23—C22—C25—C26	55.16 (13)
C10—N4—C12—C13	−134.62 (14)	C28—C22—C25—C26	178.40 (10)
C10—N4—C12—C24	47.87 (19)	C22—C25—C26—C27	−179.98 (10)
C24—C12—C13—C14	1.44 (19)	C21—C22—C28—C29	51.69 (14)
N4—C12—C13—C14	−176.07 (12)	C23—C22—C28—C29	−60.50 (14)
C12—C13—C14—C15	−0.45 (18)	C25—C22—C28—C29	175.71 (10)
C13—C14—C15—C23	−0.48 (17)	C22—C28—C29—C30	179.50 (11)
C13—C14—C15—C16	178.88 (12)		

### Hydrogen-bond geometry (Å, º)

**Table:**

*D*—H···*A*	*D*—H	H···*A*	*D*···*A*	*D*—H···*A*
N4—H4···N2	0.88	2.55	2.8567 (17)	101
C5—H5*B*···N1	0.99	2.60	3.212 (2)	120
C20—H20···N3^i^	0.95	2.55	3.4577 (16)	161

Symmetry code: (i) *x*, *y*, *z*+1.
